# Albumin-To-Alkaline Phosphatase Ratio as a New Early Predictive Marker of Axillary Response in Breast Cancer Patients Undergoing Neoadjuvant Chemotherapy: A Pilot Study

**DOI:** 10.3390/medicina60111767

**Published:** 2024-10-28

**Authors:** Rahel Felicia Mirjam Schmidt, Yves Harder, Lorenzo Rossi, Paola Canino, Simone Schiaffino, Arianna Calcinotto, Ulrike Perriard, Rossella Graffeo, Roberta Decio, Claudia Canonica, Marco Cuzzocrea, Ammad Ahmad Farooqi, Giorgia Elisabeth Colombo, Mirjam Diller, Nickolas Peradze, Andrea Papadia, Alberto Pagnamenta, Maria Luisa Gasparri

**Affiliations:** 1 Faculty of Biomedical Science, Università della Svizzera Italiana (USI), 6900 Lugano, Switzerland; rahel.schmidt@usi.ch (R.F.M.S.); simone.schiaffino@eoc.ch (S.S.); arianna.calcinotto@ior.usi.ch (A.C.); andrea.papadia@eoc.ch (A.P.); alberto.pagnamenta@eoc.ch (A.P.); 2 Department of Plastic, Reconstructive and Aesthetic Surgery and Hand Surgery, Centre Hospitalier Universitaire Vaudois (CHUV), 1005 Lausanne, Switzerland; yves.harder@chuv.ch; 3 Faculty of Biology and Medicine, University of Lausanne (UNIL), 1011 Lausanne, Switzerland; 4 Centro di Senologia della Svizzera Italiana (CSSI), Ente Ospedaliero Cantonale (EOC), 6900 Lugano, Switzerland; lorenzo.rossi@eoc.ch (L.R.); paola.canino@eoc.ch (P.C.); ulrike.perriard@eoc.ch (U.P.); rossella.graffeogalbiati@eoc.ch (R.G.); roberta.decio@eoc.ch (R.D.); claudia.canonica@eoc.ch (C.C.); marco.cuzzocrea@eoc.ch (M.C.); nickolas.peradze@eoc.ch (N.P.); 5 Istituto Oncologico della Svizzera Italiana (IOSI), Centro di Senologia della Svizzera Italiana, 6962 Lugano, Switzerland; 6 Servizio di Radio-Oncologia, Istituto Oncologico della Svizzera Italiana, Ente Ospedaliere Cantonale (EOC), Centro di Senologia della Svizzera Italiana, 6962 Lugano, Switzerland; 7 Servizio di Radiologia, Istituto Imaging della Svizzera Italiana, Ente Ospedaliere Cantonale (EOC), Centro di Senologia della Svizzera Italiana, 6962 Lugano, Switzerland; 8 Cancer Immunotherapy Lab, Institute of Oncology Research (IOR), 6500 Bellinzona, Switzerland; 9 Servizio di Istopatologia, Istituto Cantonale di Patologia, Ente Ospedaliere Cantonale (EOC), 6600 Locarno, Switzerland; 10 Dipartimento di Ginecologia e Ostetricia, Ente Ospedaliere Cantonale (EOC), Centro di Senologia della Svizzera Italiana, 6962 Lugano, Switzerland; giorgiaelisabeth.colombo@eoc.ch (G.E.C.); mirjam.diller@eoc.ch (M.D.); 11 Clinica di Medicina Nucleare, Ente Ospedaliere Cantonale (EOC), dell’Istituto Imaging della Svizzera Italiana, Ospedale Regionale di Bellinzona sede San Giovanni e Ospedale Regionale di Lugano sede Civico, 6900 Lugano, Switzerland; 12 Institute of Biomedical and Genetic Engineering (IBGE), Rashid Latif Medical College, Lahore 54000, Pakistan; farooquiammadahmad@gmail.com; 13 Clinical Trial Unit, Ente Ospedaliere Cantonale (EOC), 6900 Lugano, Switzerland

**Keywords:** albumin-to-alkaline phosphatase ratio, axillary lymphadenectomy, breast cancer, neoadjuvant chemotherapy, sentinel lymph node biopsy

## Abstract

*Background and Objectives:* The Albumin-to-Alkaline Phosphatase ratio (AAPR) is an easily applicable and cost-effective marker investigated as an outcome predictor in solid cancers. Preliminary evidence in breast cancer suggests that a low AAPR correlates with a poor response to neoadjuvant chemotherapy (NAC) in primary tumors. However, data regarding the axillary response are lacking. This study aims to evaluate whether the AAPR can predict the axillary response in initially nodal-positive (cN+) breast cancer patients undergoing NAC. *Materials and Methods*: Clinical and biochemical variables of cN+ breast cancer patients undergoing NAC were collected. Pre-NAC albumin and alkaline phosphatase serum values were utilized in the AAPR calculation. Fisher’s exact test was performed to identify differences between the two groups of patients (high and low AAPR according to the cut-off reported in the literature). The primary outcome was the nodal pathologic complete response (pCR) rate in the two groups of patients. *Results:* Nodal pCR was achieved in 20/45 (44.4%) patients. A total of 36/45 (80%) patients had an AAPR > 0.583. Among patient and tumor characteristics, the only statistically significant difference between the two groups was the axillary pCR between the low and high AAPR groups (*p*-value = 0.03, OR = 0.129, 95% CI = 0.00–0.835). *Conclusions:* This pilot study suggests that the pre-treatment AAPR might be an early predictor of axillary response in cN+ breast cancer patients undergoing NAC. This result justifies further investigation in larger prospective trials to validate this finding.

## 1. Introduction

Breast cancer is ranked among the leading causes of cancer-related mortality worldwide, affects millions of women, and thus is a major global health burden [1]. The surgical treatment in the non-metastatic setting includes two compartments, breast and axilla, in a multimodal and interdisciplinary approach. In the last decades, axillary surgery has no longer been considered a therapeutic procedure in the majority of cases, shifting towards becoming purely a staging and prognostic procedure, able to provide an indication of further therapy required.

Nearly one-third of those diagnosed with primary breast cancer has also lymph node involvement at the time of diagnosis (cN+) [2]. As for the primary breast tumor, where NAC plays a pivotal role in reducing the tumor burden and allows a surgical de-escalation from mastectomy to breast conservative surgery [3], NAC in the axillary compartment aims to provide a response that allows a minimally invasive procedure instead of a full axillary lymphadenectomy. The rate of axillary lymph node response after NAC depends on tumor subtype, and the pathologic complete response (pCR) varies between 16% in hormonal receptor-positive HER-2-negative breast cancer, 70% in triple-negative breast cancer, and up to 80% in HER-2-positive breast cancer [4]. There is no international consensus on axillary management after NAC in cN+ breast cancer. The reason that the sentinel lymph node biopsy is not universally accepted after NAC instead of axillary lymphadenectomy is due to the high false negative rate in this setting, which exceeds 10% in most international prospective validation studies [5,6,7,8,9,10,11]. In order to improve the performance of this technique, NCCN guidelines suggest collecting at least 3 sentinel lymph nodes and/or adopting more than one tracer [12]. The false negative rate (FNR) of sentinel lymph node biopsy decreases significantly with the number of sentinel lymph nodes removed (2 sentinel lymph nodes = 12% FNR; 3 sentinel lymph nodes = 4% FNR) [13]. However, the more lymph nodes we remove, the higher the morbidity. Therefore, the more lymph nodes we remove, the closer we obtain to an axillary lymphadenectomy (which is commonly defined as the removal of at least 10 nodes) both in terms of FNR and in terms of morbidity. Additionally, it is not always technically feasible to obtain at least 3 sentinel lymph nodes since even when the mapping is performed correctly, the tracer can detect a maximum of one sentinel lymph node. Even when dual tracers are adopted, the false negative rate is generally slightly above what is considered the upper acceptable limit of 10%. The selective removal of the metastatic lymph node marked before NAC associated with biopsy of the sentinel lymph node (targeted axillary dissection) represents an “in-between solution” with good surgical performance and low FNR [14]. Evidence on oncological outcomes suggests that both approaches are equally safe in this setting and provide the same prognostic information [15]. 

Today, TAD is the most common post-NAC axillary surgery in patients with cN+ disease converting to ycN0, followed by sentinel lymph node biopsy (SLNB) alone [16]. Differences in surgical performance and patients’ perspectives are currently under evaluation. The current ongoing AXSANA (EUBREAST-3) study was designed to reduce international heterogeneity and standardize guidelines [17].

Nevertheless, both in the case of sentinel lymph node biopsy and in the case of TAD, the key point for axillary surgical de-escalation remains the identification of which patients may benefit the most from NAC. Predictive models are being investigated, and so far, several independent clinical and histological predictive factors associated with nodal pathologic complete response (pCR) have been identified, with nomograms to predict axillary status after NAC in cN+ breast cancer patients proposed [3,18,19,20,21]. Clinicopathological indicators have also been integrated with laboratory markers in evaluating response to NAC [22,23,24,25]. In order to increase the accuracy of these predictive tools, additional biomarkers are needed and are, therefore, currently under investigation. 

The Albumin-to-Alkaline Phosphatase ratio (AAPR) is an easily applicable laboratory marker shown to predict pCR after NAC as well as oncologic outcomes in various solid tumors, including glioblastoma, bladder, renal, cervical, gastric, hepatic, gallbladder, and pulmonary cancer. A low AAPR is associated with a less favorable outcome and inadequate response to NAC [26]. 

In breast cancer, three retrospective studies have investigated AAPR as a predictor of response to NAC in the primary tumor and a predictor of overall survival with promising results [27,28,29]. However, none of them investigated the AAPR as a selective predictor of axillary nodal response. The aim of this pilot study is to investigate whether the AAPR can predict pCR in cN+ breast cancer patients receiving NAC.

## 2. Materials and Methods

### 2.1. Study Design

A retrospective analysis including cN+ breast cancer patients undergoing NAC has been performed. All patients have been tested for Albumin and Alkaline Phosphatase, and the AAPR has been calculated. According to the cutoff determined in literature in a primary tumor setting [27], the patients have been divided into 2 groups (above and below the cutoff). Patient and tumor characteristics and pathological responses have been analyzed between the two groups. 

### 2.2. Study Population and Ethics Approval

The population eligible for inclusion comprised patients with cN+ breast cancer treated at the Centro di Senologia della Svizzera Italiana (CSSI), Ente Ospedaliero Cantonale (EOC), between January 2016 and January 2023. The inclusion criteria were as follows: (1) clinical and pathological diagnosis of breast cancer; (2) initially cN+ disease; (3) non-metastatic (M0) disease; (4) NAC according to the standard of care followed by documented breast and axillary surgery (TAD/SLND in case of clinical and radiological conversion from cN+ to cN0; ALND in case of no nodal response from NAC); (5) pre-treatment serum albumin and alkaline phosphatase levels; (6) not objection letter recipients patients with only ITCs at the time of axillary staging were not included; micrometastases were considered in the group of no pathologic complete response, given their subsequent required treatment (ALND) as for macrometastases, according with NCCN recommendation [12]. Patients with incomplete information in their medical records for the outcomes extracted were excluded from the analysis. This study was reviewed and approved by the Ethics Committee of Ticino (BASEC ID 2020-01121). 

### 2.3. Data Collection

The medical records of eligible patients were analyzed, and data were extracted from the hospital database. Patient characteristics retrieved for further analysis included age, tumor size, nodal status, histological subtype, histological grade, hormone receptor (HR) status, HER2 status, Ki67 expression, pathological analysis on the primary tumor and axillary lymph node, as well as the pre-treatment serum albumin and alkaline phosphatase levels.

### 2.4. Statistical Analysis

The continuous variables (age and AAPR) were dichotomized by determining a threshold. The threshold for age was determined by the median value (51, range 24–87). The threshold utilized for the AAPR cut-off was 0.583, according to the results from Qu et al. [27]. AAPR values below the threshold were given a score of 0, and those above the threshold were given a score of 1.

Fisher’s exact test was performed to assess the differences in patients and tumor’s characteristics between patients with high and low AAPR. The odds ratio (OR) with the corresponding 95% confidence intervals (CI) were presented. A *p*-value < 0.05 was considered significant. All statistical analyses were performed using R software Version 4.2.2 (R Foundation for Statistical Computing, Vienna, Austria) and IBM SPSS Statistics Version 28 (IBM Corp., Armonk, NY, USA).

## 3. Results

From January 2016 to January 2023, seventy-eight patients met the inclusion criteria, of which 33 had incomplete medical records and thus were excluded. A total of 45 patients were analyzed in this pilot study (Table 1). 

The median age of the included participants was 51 years, with a range between 24 and 87 years. The most frequent breast cancer histotype was invasive ductal carcinoma (n = 39, 86.7%). Thirty patients (68.9%) had a clinical tumor stage (cT) of T1–2 at diagnosis, while the other third had a clinical stage of T3–4 (n = 15, 33.3%). Thirty-one patients (68.9%) were cN1, while the others were cN2 or cN3 (n = 14, 31.1%). A histological grade of 1–2 was observed in 10 patients (22.2%), whereas 35 patients (77.8%) were diagnosed with a histological grade of 3. Hormone receptor status was positive in 33 patients (73.3%), while there was no expression of either estrogen or progesterone receptors in 12 patients (27.7%). Twenty-four patients (53.3%) had a positive HER2 status. Only two patients had Ki67 expression below 20% (4.4%). Primary tumor pCR was achieved in 22 patients (48.9%) and nodal pCR in 20 patients (44.4%). 

Analyzing the results of Fisher’s exact test and the odds ratios of the underlying data found that nodal pCR was significantly lower in the AAPR-low group compared to the AAPR-high group (OR 0.129, 95% CI 0.005–0.835, Fisher’s exact test *p* = 0.03). There was no significant difference in all other clinicopathological characteristics between the AAPR-low and AAPR-high groups. Detailed results of the analysis are presented in Table 1.

## 4. Discussion

Over the last few years, there has been a lot of progress in finding a desirable balance to minimize surgical morbidity while having an optimal oncological outcome. In the case of breast cancer patients with clinical axillary lymph node metastases, the approach has shifted from “not leaving a single potentially affected lymph node in the axilla” (complete lymphadenectomy) to a “pick and choose” approach (sentinel lymph node biopsy/targeted axillary dissection). Ideally, complete axillary lymphadenectomy should be confined to selected patients. Complete axillary lymphadenectomy is associated with higher morbidity as compared to TAD and sentinel lymph node biopsy [30]. Side effects of radical axillary management include paresthesia in the axillary region down to the ipsilateral arm, restriction in the range of motion, as well as lymphedema of the ipsilateral arm caused by dysfunctional lymphatic drainage [19]. The latter is the most feared complication, as the swelling may lead to skin damage and an inability to move, which in turn favors infection (cellulitis) and, ultimately, sepsis [20]. Additionally, lymphedema can also affect subsequent loco-regional treatment, such as radiation therapy. Different options have been developed in order to minimize this adverse event; however, the result is still suboptimal for most patients [31,32,33].

Following NAC, the systematic removal of lymph nodes from the axilla, usually level I and II, sometimes also including level III, is a surgical procedure confined only to patients who still harbor metastatic disease. In cN+ breast cancer patients converting to ycN0, axillary surgical treatment consists of TAD or sentinel lymph node biopsy in most institutions worldwide. Unfortunately, a considerable percentage (27.6–83.5%, depending on cancer subtype) of cases do not undergo a complete response in the axilla with NAC (ycN+), which is revealed only after NAC by clinical and radiological assessment [3]. In these cases, the recommended surgical management of the axilla remains axillary lymph node dissection. 

A priori identification of breast cancer patients who will benefit the most from NAC is of high interest for the patient selection process. In cN+ breast cancer patients undergoing NAC with the sole intent of a subsequent de-escalation, presenting with a predicted low rate of response to NAC at diagnosis, surgery could be performed upfront without further delay as the surgical procedure remains the same in the case of axillary clinical involvement. Chemotherapy could be given in the adjuvant setting rather than in the neoadjuvant setting to avoid delaying surgical therapy in this case. 

Tools permitting a reliable calculation of the rate of response to NAC at diagnosis are currently under investigation. Predictive models have been developed to identify breast cancer patients likely to have their lymph node status converted from positive to negative after NAC. Clinical and radiological profiling, as well as pre-treatment laboratory indicators of response, are currently under investigation in order to identify the subset of patients that will benefit the most from NAC at the time of diagnosis.

Recently, in the same way this study was designed, we demonstrated in a larger series that a low neutrophil–lymphocyte ratio and a low pan-immune-inflammation value predict axillary pCR in cN+ in breast cancer patients undergoing NAC [34].

The current pilot study has shown that there is a significant difference in the rate of axillary pCR to NAC in initially cN+ breast cancer patients with low AAPR compared to high AAPR. 

The AAPR itself includes two laboratory markers associated with nutritional status and inflammation. An obvious advantage of laboratory predictors such as the AAPR is that they are easily applicable since the involved laboratory markers are part of standard venous blood sampling and, thus, do not need further invasive or cost-intensive measures. 

Albumin is an acute-phase protein synthesized in the liver, which can, therefore, provide information on systemic inflammation, nutritional status, and hepatic function [35]. Hypoalbuminemia in all disease processes correlates with a poor prognosis, higher mortality, and surgical complications. Chronically ill patients, such as cancer patients, often have low albumin levels. The theory that low albumin in chronic illness is caused by TNF and IL-6 (acute-phase proteins) decreasing the synthesis of albumin is supported by various animal studies. However, the contrary was observed in human studies, as albumin production was increased in chronically ill patients, leading to the hypothesis that a higher clearance rate leads to hypoalbuminemia in these patients. The exact pathophysiology of hypoalbuminemia is not entirely understood [36].

On the other hand, alkaline phosphatase is produced by various tissues such as the liver, the bile duct, kidney, and bone, making it a marker for cholestasis, bone turnover, and notably for tumor growth and metastasis [37,38]. 

Both laboratory markers can be altered in patients with a malignant tumor due to a tumor-induced pro-inflammatory state (albumin) and the direct effects of invasive tumor growth itself (alkaline phosphatase). Hence, combining both makers to form the AAPR has been shown to be a more accurate indicator of tumor behavior than albumin or alkaline phosphatase alone. A decrease in the ratio correlates significantly with an adverse effect on prognosis, including disease-free survival, progression-free survival, and cancer-specific survival [27].

In breast cancer, only three retrospective studies have evaluated the potential role of AAPR as an outcome predictor (Table 2) [27,28,29]. One of these assessed the role of AAPR as a predictor of response to NAC in the primary tumor, regardless of axillary involvement [27], while the others investigated AAPR as a predictor of overall survival [27,28,29]. These studies defined the AAPR cut-off value using a receiver operating characteristic curve (ROC curve) analysis to stratify patients into a low-AAPR group and a high-AAPR group (Table 2). 

With a cut-off value of 0.583, AAPR has been shown to be a predictor of pCR in the primary tumor (*p* = 0.007, OR = 2.228, 95% CI 1.246–3.986) [27]. A nomogram for the prediction of the oncological outcome has been created based on four inflammatory-nutritional markers, including AAPR (*p* = 0.043 (HR = 0.447, 95% CI = 0.205–0.976) [28]. In a multivariate analysis, the AAPR has been demonstrated to be an independent variable affecting the overall survival in breast cancer patients (*p* = 0.043, HR = 0.447; 95% CI 0.205–0.976) [29].

In our study cohort, the AAPR threshold of 0.583 was applied based on the data reported by Qu et al. [27]. This is for two reasons: firstly, our sample was too small to conduct a ROC analysis, and secondly, the cut-off value proposed by Qu et al. [27] is more applicable to our cohort as it is the only study evaluating the role of AAPR in predicting tumor pCR as a primary endpoint in breast cancer, as opposed to overall survival. 

This pilot study has some limitations to consider when interpreting the results. The small cohort leads to an increased risk of type II error, and this limitation meant that it was not possible to conduct a multivariate logistic regression analysis. For the same reason, we can assume that the sample size was not able to determine a significant result in the evaluation of primary tumor response. Additionally, the retrospective nature of this study may introduce more bias than prospective study methods.

On the other side, the strength of this study is that a statistically significant difference regarding nodal pCR has been found, despite the small sample. 

The presented results provide a novel insight into the suitability of AAPR as a predictor for pCR in the axilla, an application that has not previously been investigated. Additionally, considering that pCR in axillary lymph nodes after NAC can be considered an early surrogate marker of the long-term outcome, ref. [39], the identification of pre-NAC predictors of axillary response can offer timely additional information on the oncological outcome. It means that in the future, a patient’s laboratory phenotype, including inflammatory markers such as neutrophil–lymphocyte ratio and pan-immune-inflammatory value, could identify a population of newly diagnosed breast cancer patients with poor prognosis.

## 5. Conclusions

AAPR may represent a novel, easily applicable, and cost-effective predictor for axillary nodal pCR in cN+ breast cancer patients undergoing NAC. Further large prospective trials are required to validate this preliminary result and incorporate it into new predictive models and nomograms. 

## Figures and Tables

**Table 1:** Patients and tumor’s characteristics.

	Total Number, 45 (100%)	AAPR < 0.583, n = 9	AAPR ≥ 0.583, n = 36	*p*-Value Fisher’s Exact T.	OR with 95% CI
Age (median = 51)					
<median	23 (51.1%)	3 (6.7%)	20 (44.4%)		
≥median	22 (48.9%)	6 (13.3%)	16 (35.6%)		
				0.284	0.416 (0.073; 1.906)
Histological type					
ductal	39 (86.7%)	8 (17.8%)	31 (68.9%)		
other	6 (13.3%)	1 (2.2%)	5 (11.1%)		
				1.000	1.175 (0.149; 34.343)
cT					
T1–2	30 (66.7%)	4 (8.9%)	26 (57.8%)		
T3–4	15 (33.3%)	5 (11.1%)	10 (22.2%)		
				0.135	0.319 (0.063; 1.493)
cN					
N1	31 (68.9%)	6 (13.3%)	25 (55.6%)		
N2–3	14 (31.1%)	3 (6.7%)	11 (24.4%)		
				1.000	0.867 (0.183; 5.014)
Histological grade					
1–2	10 (22.2%)	1 (2.2%)	9 (20%)		
3	35 (78.8%)	8 (17.8%)	27 (60%)		
				0.659	0.421 (0.015; 2.897)
Hormone receptor status					
negative	12 (26.7%)	2 (4.4%)	10 (22.2%)		
positive	33 (73.3%)	7 (15.6%)	26 (57.8%)		
				1.000	0.778 (0.093; 4.069)
HER2 status					
negative	21 (46.7%)	5 (11.1%)	16 (35.6%)		
positive	24 (53.3%)	4 (8.9%)	20 (44.4%)		
				0.713	1.537 (0.337; 7.473)
Ki67 expression					
<20	2 (4.4%)	0 (0%)	2 (4.4%)		
≥20	43 (95.6%)	9 (20%)	34 (75.6%)		
				1.000	NA
pCR in primary tumor					
ypT0	22 (48.9%)	2 (4.4%)	20 (44.4%)		
ypT ≥ 1	23 (51.1%)	7 (15.6%)	16 (35.6%)		
				0.135	0.246 (0.030; 1.227)
pCR in lymph nodes					
ypN0	20 (44.4%)	1 (2.2%)	19 (42.2%)		
ypN ≥ 1	25 (55.6%)	8 (17.8%)	17 (37.8%)		
				0.030	0.129 (0.005; 0.835)

**Table 2:** Overview of all studies investigating AAPR as a predictor for OS and/or pCR in breast cancer patients.

	Study Population	Study Design	AAPR Cut-Off	Cut-Off Definition	Primary Endpoint	Findings
Long et al., 2019 [29]	746	retrospective	0.525	ROC curve	OS	Low AAPR group: 5-year OS rate 80.16%
						High AAPR group: 5-year OS rate 92.66%
Qu et al., 2021 [27]	780	retrospective	0.583	ROC curve	T pCR	Univariate analysis: pCR correlated with AAPR (*p* = 0.03)
						Multivariate analysis: AAPR correlated with pCR (*p* = 0.03)
Hua et al., 2022 [28]	1259	retrospective	0.576	C-index and ROC curve	OS	Construction of INPS with Lasso Cox regression included
						NLR, MLR, PNI, and AAPR multivariate Cox regression: INPS independent indicator of OS (HR = 0.51; 95% CI: 0.35–0.75, *p* < 0.001)

## Data Availability

The data presented in this study are available on request from the corresponding author due to confidentiality issues bound by the ethics approval.
